# The multifaceted involvement of exosomes in tumor progression: Induction and inhibition

**DOI:** 10.1002/mco2.49

**Published:** 2021-07-01

**Authors:** Wen‐Jie Gu, Yi‐Wen Shen, Li‐Jun Zhang, Hong Zhang, Dale G. Nagle, Xin Luan, San‐Hong Liu

**Affiliations:** ^1^ Institute of Interdisciplinary Integrative Medicine Research Shanghai University of Traditional Chinese Medicine Shanghai China; ^2^ Department of BioMolecular Sciences and Research Institute of Pharmaceutical Sciences School of Pharmacy University of Mississippi University Mississippi USA

**Keywords:** biomarker, cancer, drug delivery, exosome, microRNAs

## Abstract

As key performers in intercellular communication, exosomes released by tumor cells play an important role in cancer development, including angiogenesis, cancer‐associated fibroblasts activation, epithelial‐mesenchymal transformation (EMT), immune escape, and pre‐metastatic niche formation. Meanwhile, other cells in tumor microenvironment (TME) can secrete exosomes and facilitate tumor progression. Elucidating mechanisms regarding these processes may offer perspectives for exosome‐based antitumor strategies. In this review, we mainly introduce the versatile roles of tumor or stromal cell derived exosomes in cancer development, with a particular focus on the biological capabilities and functionalities of their diverse contents, such as miRNAs, lncRNAs, and circRNAs. The potential clinical application of exosomes as biomarkers in cancer diagnosis and prognosis is also discussed. Finally, the current antitumor strategies based on exosomes in immunotherapy and targeted delivery for chemotherapeutic or biological agents are summarized.

## Graphical abstract

Exosomes released by tumor cells and tumor microenvironment‐associated cells play a pivotal role in cancer initiation and progression. Exosomes facilitate cancer development in multiple aspects, including angiogenesis, cancer‐associated fibroblasts activation, immune escape, epithelial‐mesenchymal transformation, pre‐metastasis niche formation, and drug resistance.

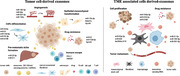

## 1 INTRODUCTION

Exosomes, the nanosized (40‐160 nm) subgroups of extracellular vesicles (EVs), were initially believed to be the platelet dust in plasma.^1^, ^2^ Exosomes are generated in the maturation process of endosomes to multivesicular bodies (MVBs). In early endosomes, molecular materials such as proteins, RNA, and DNA, are collected and further processed. Then the late endosomes mature into MVBs, and intraluminal vesicles (ILVs) are formed. Eventually, MVBs can fuse with the plasma membrane, and ILVs are released as exosomes into extracellular space.^3^ In the past decades, studies have unraveled that exosomes are important mediators of intercellular communication and are involved in a diverse range of biological processes with the varying cargos inside, including lipids, proteins, nucleic acids, and metabolites.^2^ When exosomes are released into the extracellular space, they can reach recipient cells and deliver cargos, exerting functional effects and inducing related phenotypic changes.^4^

**FIGURE 1:**
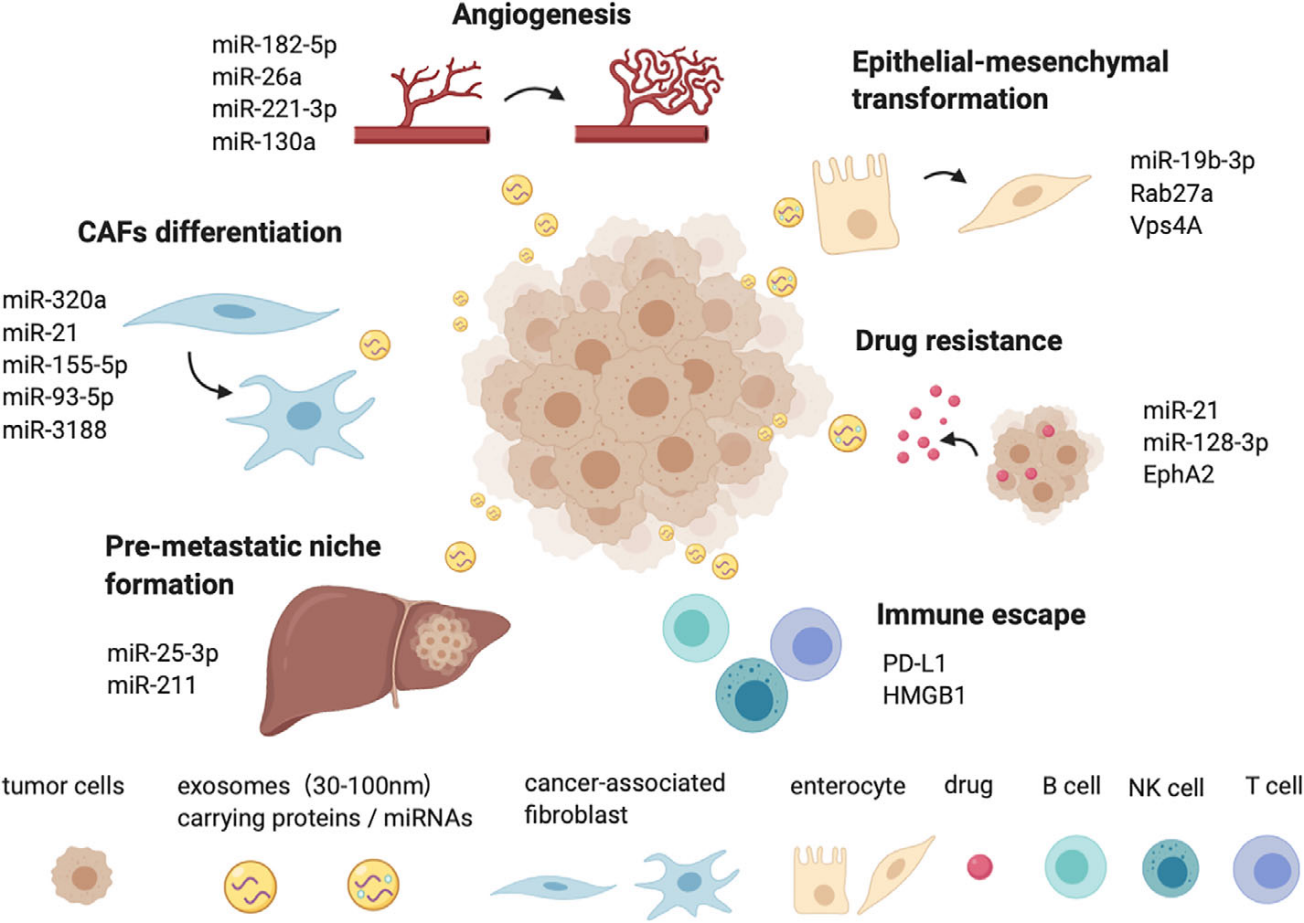
The functions of tumor cell‐derived exosomes in cancer development. Tumor cell‐derived exosomes carrying biological molecules, such as miRNAs, proteins, and nucleic acids, are involved in various aspects of tumor progression, including angiogenesis, cancer‐associated fibroblasts activation, immune escape, epithelial‐mesenchymal transformation, pre‐metastasis niche formation, and drug resistance

**FIGURE 2:**
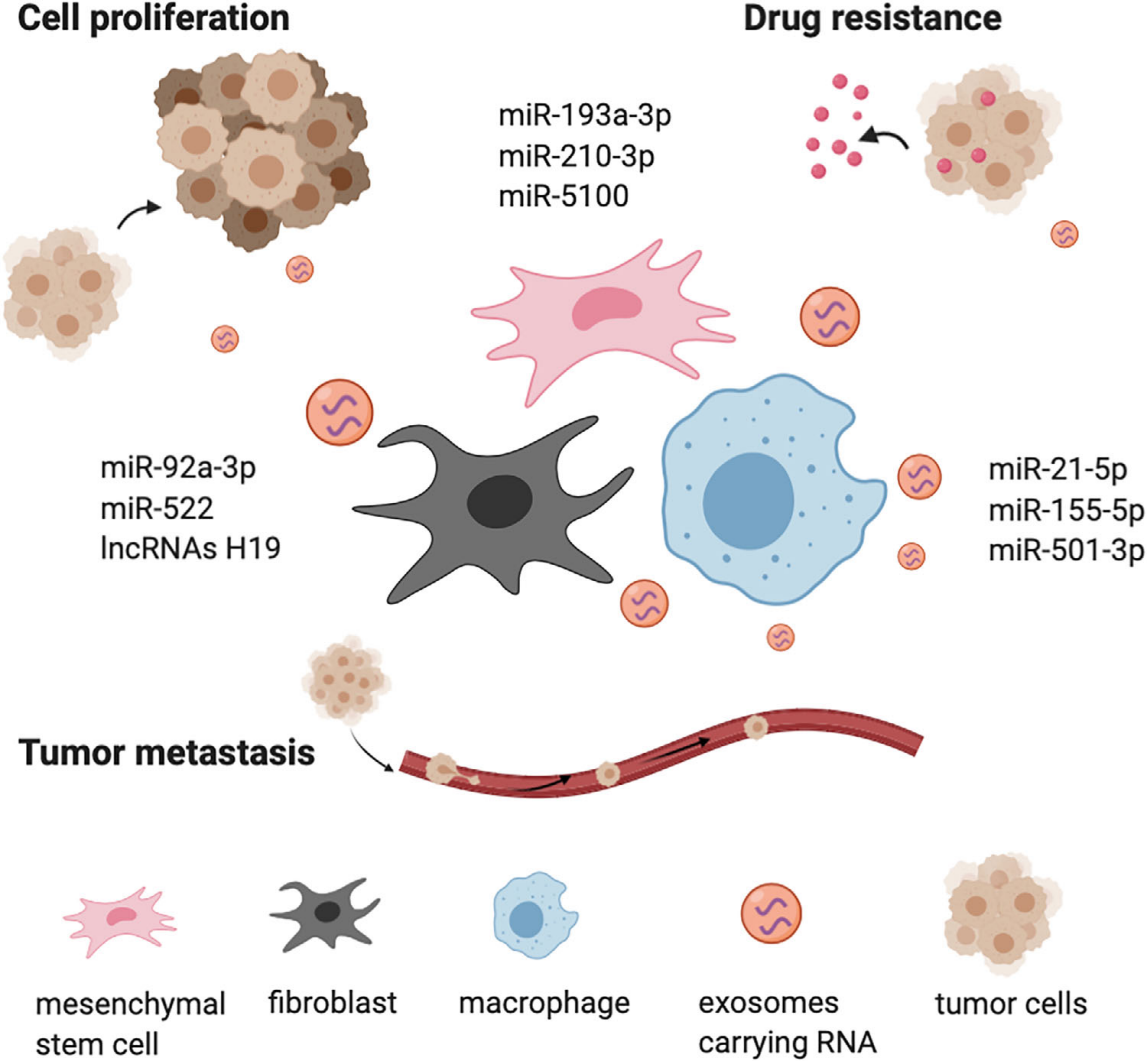
The functions of exosomes from other cells in TME in cancer development. Exosomes from other cells in TME, such as mesenchymal stem cells, cancer‐associated fibroblasts and macrophages can promote cell proliferation, drug resistance, and tumor metastasis, thus facilitating tumor progression

Exosomes are transferred between cells and exhibit multi‐faceted physiological and pathological function. Exosomes can mediate the cell‐to‐cell communication and maintain the normal bioactivities of recipient cells through the transportation of cargos within extracellular space.^5^ On the other hand, exosomes also play a critical role in the progress of some diseases, especially as the transmitters in tumor microenvironment (TME).^6^, ^7^ It has been demonstrated that cancer cells exhibited the significantly increased exosomes secretion compared with normal cells with profound influences on tumor progression and metastasis.^6^, ^7^ For example, exosomes derived from tumor cells can alter the properties of normal cells by carrying oncogenic materials and initiate phenotypic changes in TME, thus progressing cancer development.^8^, ^9^ Emerging evidences have also proved that exosomes are responsible for the tumor cell expansion, metabolic activity remodeling, tumor angiogenesis, immunosuppressive TME, and acquired drug resistance (Figures 1 and 2).^10^ These studies urge the need of corresponding regarding these revealed mechanisms.

**FIGURE 3:**
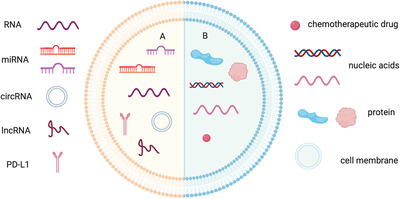
Application of exosomes in cancer diagnosis and therapy. A, Exosomes containing specific RNAs and proteins can be utilized as noninvasive biomarkers for cancer diagnosis and prognosis. B, Exosomes can serve as nanocarriers for drug delivery and can be applied in immunotherapy as cancer therapeutic vaccine

Research have shown that exosomes are recognized as important transporters in cancer and exhibit promising prospects for cancer diagnosis (Figure 3).^11^ Recently, the applications of exosomes have been focused on biomarkers in diagnosis, underlying functions and mechanisms, and drug delivery systems owing to its unique natural features.^12^, ^13^ In this review, we focus on the present studies and the underlying mechanisms of exosomes, especially regarding the involvement in TME. Additionally, we conclude the application exosomes in tumor diagnosis and therapy.

## 2 ROLE OF TUMOR CELL‐DERIVED EXOSOMES FOR TUMOR PROGRESSION

Exosomes function variably according to the origin of cells. Tumor cells‐derived exosomes (TCDEs) contribute to the tumor progression through the induction of tumor cell proliferation, invasion, and migration, and are also proved to be the key attributes of tumorigenicity and TME maintenance. Targeting TME is widely accepted as a promising strategy to effectively suppress tumor progression, and six important aspects of involvement in tumor progression regarding tumor cell‐released exosomes have been summarized as follows (Figure 1 and Table 1).

**TABLE 1:** Role of tumor cell‐derived exosomes for tumor progression

Cell type	Exosomal cargo	Function	Mechanism	Reference
**Angiogenesis**				
Papillary thyroid cancer cells	miR‐21‐5p	Increase angiogenesis	Target and suppress TGFBI and COL4A1	^17^
Ovarian cancer cells	miR‐205	Induce angiogenesis and promote metastasis	Via the PTEN‐AKT pathway	^18^
Lung cancer cells	miR‐23a	Increase angiogenesis and vascular permeability	Target prolyl hydroxylase and tight junction protein ZO‐1	^20^
Glioblastoma cells	miR‐182‐5p	Promote angiogenesis	Target kruppel‐like factor 2 and 4	^21^
Glioblastoma cells	miR‐26a	Promote angiogenesis	Activation of the PI3K/Akt signaling pathway by targeting PTEN.	^22^
Cervical squamous cells	miR‐221‐3p	Promote angiogenesis	Target THBS2	^23^
Gastric cancer cells	miR‐130a	Activate angiogenesis	Target C‐MYB	^16^
Gastric cancer cells	miR‐155	Promote angiogenesis	Target forkhead box O3	^24^
Gastric cancer cells	miR‐135b	Promote angiogenesis	Inhibit FOXO1 expression	^25^
Gastric cancer cells	miR‐23a	Promote angiogenesis	Target PTEN	^26^
Gastric carcinoma cells	miR‐155	Promote angiogenesis	Target the c‐MYB/ VEGF axis	^27^
Colorectal cancer cells	miR‐183‐5p	Promote angiogenesis	Regulation of FOXO1	^28^
Colorectal cancer cells	miR‐1229	Promote angiogenesis	Target HIPK2	^29^
Hepatocellular carcinoma cells	Angiopoietin‐2	Induce angiogenesis	Activate the AKT/eNOS and AKT/β‐catenin pathways in HUVECs	^30^
Hepatocellular carcinoma cells	LOXL4	Promote angiogenesis and cell migration	Paracrine transfer mechanism; activate FAK/Src pathway	^31^
Colorectal cancer cells	miR‐590‐5p	Inhibit angiogenesis and metastasis	Regulate NF 90/VEGFA Axis	^32^
Menstrual mesenchymal stem cells	/	Inhibit angiogenesis and tumor growth	Induce endothelial cell death, modulate VEGF secretion	^33^
Liver stem cells	miR‐15a miR181b miR320c miR‐874	Inhibit angiogenesis	Downregulation of FGF1 and PLAU	^34^
Breast cancer cells	miR‐23b miR‐27b miR‐320b	Inhibit angiogenesis	Decrease the expression of PLAU, AMOTL1, NRP1, ETS2	^35^
**Cancer‐associated fibroblast**				
Hepatocellular carcinoma cells	miR‐21	Convert hepatocyte stellate cells to cancer‐associated fibroblasts, contribute to tumor progression	miRNA‐21 targets PTEN, leading to activation of PDK1/AKT signaling; CAF secrete VEGF, MMP2, MMP9, bFGF, and TGF‐β	^39^
Melanoma cells	miR‐155‐5p	Induce reprogramming of fibroblasts into CAFs miR‐155 trigger the proangiogenic switch of CAFs promote the expression of VEGFa, FGF2, and MMP9	Via the SOCS1/JAK2/STAT3 signaling pathway	^40^
Oral cancer cells	miR‐34a‐5p	Confer aggressiveness Induce EMT and promote cancer cells metastasis	Via the AKT/GSK‐3β/β‐catenin/Snail signaling cascade	^41^
Colorectal cancer cells	miR‐93‐5p	Higher miR‐93‐5p leads to radioresistance and increase the tumor growth	Downregulation of FOXA1 and upregulation of TGF‐β3	^42^
**Immune escape**				
Melanoma cells	PD‐L1	Increased PD‐L1 suppresses CD8+T cells and facilitates tumor growth	Through stimulation with interferon‐γ (IFN‐γ)	^48^
Non‐small cell lung cancer cells	PD‐L1	Reduce cytokine production and induce apoptosis in CD8^+^ T cells	Inhibit IFN‐γ secretion and impair immune functions	^50^
Hepatocellular carcinoma cells	HMGB1	Foster immune evasion and promote the advanced disease stage	Activate B cells and promote TIM‐1^+^ Breg expansion via TLR 2/4 and MAPK signaling pathways	^52^
Melanoma cells	miR‐3187‐3p miR‐498 miR‐122 miR149 miR‐181a/b	Downregulate T‐cell responses	Through decreased TCR signaling, diminished cytokine and granzyme B and TNFα secretion	^53^
Chronic lymphocytic leukemia monocytes	HY4	Release CCL2, CCL4, and IL‐6, and induce PD‐L1 expression, contribute to cancer‐related inflammation and concurrent immune escape	Through TLR7 signaling	^54^
**Epithelial‐mesenchymal transformation**				
Hypoxic bone marrow‐derived mesenchymal stem cells	miR‐193a‐3p miR‐210‐3p miR‐5100	Induce EMT and promote tumor cell invasion	Activation of STAT3 signaling	^55^
Clear cell renal cell carcinoma cells	miR‐19b‐3p	Induce EMT and confer organotropism	Decreased PTEN, CD103^+^	^56^
Melanoma cells	Decreased let‐7	Promote phenotype switching; Initiate EMT and promote metastasis	Through paracrine/autocrine signaling; Activation of MAPK pathway	^57^
Hepatocellular carcinoma cells	Rab27a blockade	Elicit EMT and facilitate the tumor progression	Through MAPK/ERK pathway	^58^
Hepatocellular carcinoma cells	/	Inhibit EMT	Vps4A mediates the plasma membrane localization and decreases exosome‐released β‐catenin	^60^
Mesenchymal stem cells	/	Reduce CAFs and suppress EMT, induce angiogenesis and maintain vascular homeostasis	/	^62^
Colorectal cancer cells	miR‐92a‐3p	Lead to EMT, promote metastasis and chemoresistance	Activation of Wnt/β‐catenin pathway and inhibition of mitochondrial apoptosis	^63^
Breast cancer cells	miR‐181d‐5p	Promote EMT	Downregulation of HOXA5 and CDX2	^64^
**Pre‐metastatic niche**				
Pancreatic ductal adenocarcinoma cells	Migration inhibitory factor (MIF)	Induce pre‐metastatic niche formation and metastasis	Driven by TGFβ‐signaling, FN deposition, and recruitment of BM‐derived macrophages	^67^
Colorectal cancer cells	miR‐25‐3p	Promote angiogenesis and vascular permeability, induce vascular leakiness and enhance metastasis	Regulate the expression of VEGFR2, ZO‐1, occludin, and Claudin5 by targeting KLF2 and KLF4	^68^
Lung epithelial cells	RNA	Initiate neutrophil recruitment and lung metastatic niche formation	Activate toll‐like receptor 3	^69^
Melanoma cells	Pigment epithelium‐derived factor (PEDF)	Loss of PEDF enables immunosuppression and abrogates the immune clearance of cancer cells metastasis	PEDF alerts host immune system, innate immune responses	^71^
**Drug resistance**				
Gastric cancer cells	miR‐21	Reduce cisplatin chemosensitivity and suppress apoptosis	MiR‐21 transfer enhances activation of PI3K/AKT signaling pathway by downregulation of PTEN	^76^
Advanced colorectal cancer cells	miR‐128‐3p	Suppress EMT and increase intracellular oxaliplatin accumulation	Negative regulation of Bmi1 and MRP5	^77^
Glioblastoma cells	miR‐15a	Enhance chemosensitivity to temozolomide	Target XRCC4	^78^
Glioblastoma cells	miR‐1238	Confer temozolomide chemoresistance	Target the CAV1/EGFR pathway	^79^
Multidrug‐resistant hepatocellular carcinoma cells	miR‐32‐5p	Induce multidrug resistance	Target PTEN via the PI3K/Akt pathway	^80^
Pancreatic cancer cells	EphA2	Transmit chemoresistance	/	^81^
Ovarian cancer cells	/	Increase cisplatin efflux, augmenting metastasis and chemotherapy resistance	Increased exosome secretion through regulation of Rab proteins by STAT3	^82^
Ovarian cancer cells	miR‐1246	Acquire chemoresistance	Modulate Cav1and p‐gp interaction	^83^

Symbol “/” represents the unknown cargo.

### 2.1 TCDE and angiogenesis

Angiogenesis is a crucial part which shows a profound influence on promoting tumor progression, consequently leading to poor prognosis. In the preexisting vascular network, new blood vessels are formed by either endothelial cells mediated‐sprouting angiogenesis or interstitial tissues‐expanded intussusceptive angiogenesis.^14^ Besides, the secretion of pro‐angiogenic factors by tumor cells triggers the creation of this disorganized, immature, and permeable vascular network, promoting cancer cell invasion and impeding the tumor‐killing action of immune cells.^15^

Surprisingly, exosomes show great potential in inducing either pro‐ or anti‐angiogenic signaling through the information delivery to endothelial cells. Additionally, exosomes adjust their cargo composition to fine tune the process of blood vessel formation in response to changes in TME.^14^ Contents of exosomes, especially miRNAs potentiate this process, and multiple studies have validated the role of exosomal miRNA in the activation of angiogenesis.^16^ TCDEs are identified to trigger angiogenesis in papillary thyroid cancer,^17^ ovarian cancer,^18^ breast cancer,^19^ and lung cancer^20^ through exosome‐mediated miRNA signaling. Under hypoxic conditions, glioblastoma cells derived‐exosomes carrying miR‐182‐5p promote tumor angiogenesis and increase vascular permeability, which are supportive for microenvironment.^21^ Consistently, glioma stem cells‐derived exosomal miR‐26a enhances angiogenesis of microvessel endothelial cells in glioma.^22^ In cervical squamous cell carcinoma (CSCC), CSCC cell‐secreted exosomal miR‐221‐3p was found to enhance the angiogenesis in the transfer to vessel endothelial cells targeting THBS2.^23^ In gastric cancer (GC), TCDEs carrying miR‐130a,^16^ miR‐155,^24^ miR‐135b,^25^ miR‐23a,^26^ and miR‐155^27^ were investigated to exert identical effect on promotion of angiogenesis through varying mechanisms. In colorectal cancer (CRC), exosomal miR‐183‐5p induces angiogenesis by regulation of FOXO1,^28^ and miR‐1229 promotes angiogenesis by targeting HIPK2.^29^ In hepatocellular carcinoma (HCC), exosome‐mediated secretion of Angiopoietin‐2‐induced angiogenesis^30^ and exosomal lysyl oxidase like 1 (LOXL) promotes cell invasion and metastasis.^31^ Most of TCDE‐related miRNAs exhibit angiogenic promotion effect, however, some exosomes are the opposite. miR‐590‐5p acted as an anti‐onco‐miR which was found to inhibit angiogenesis and metastasis through the regulation of nuclear factor (NF) 90/VEGFA axis.^32^ Several stem cells, including menstrual mesenchymal stem cells (MSCs) and liver stem cells, have been identified as inhibitors of angiogenesis, and secreted exosomes loading miRNA are detected to suppress angiogenesis and tumor growth.^33^, ^34^ In breast cancer, researchers discovered that an antiangiogenic agent, docosahexaenoic acid (DHA), triggered the secretion of exosomes and the expression level of miRNAs including miR‐23b, miR‐27b, and miR‐320b, which inhibited tube formation of endothelial cells by decreasing the expression of their respective pro‐angiogenic target genes, including PLAU, AMOTL1, NRP1, and ETS2.^35^

### 2.2 TCDE and cancer‐associated fibroblasts

Cancer‐associated fibroblasts (CAFs), a kind of stromal cells, form a part of TME and play an important role in the regulation of the tumor progression.^36^ Based on the demonstrated presence of CAFs in malignancy, Paggetti et al unveiled the presence of CAFs in chronic lymphocytic leukemia (CLL) lymph node. What's more, AKT serine/threonine kinase 1 (AKT) and NF‐κB in stromal cell were demonstrated to be essential for induction of the inflammatory phenotype. It is worth noting that, the uptake of cancer exosomes by stromal cells elicits the inflammatory effects which contribute to the CAFs transition.^37^

Ringuette et al determined the fibroblast differentiation into CAFs through transforming growth factor beta 1 (TGF‐β) via bladder cancer (BC) cells‐derived exosomes, suggesting exosomal TGF‐β can be considered as a novel molecular mechanism involved in CAF activation in tumor.^38^ Previous studies also have shown that exosomes act as a messenger between the communication/crosstalk of tumor cells and CAFs. Therefore, a deep understanding of this interaction may make contribution to the cancer therapy.

Exosome‐secreted miRNAs exert the inducing progression effects on tumors. For example, HCC‐derived exosomal miRNA‐21 induces tumor progression by activation of hepatic stellate cells into CAFs, demonstrating the role of exosome mediator.^39^ Another case in melanoma shows that exosomal miR‐155‐5p induces CAF proangiogenic function via suppressor of cytokine signaling 1 (SOCS1)/Janus kinase 2 (JAK2)/signal transducer and activator of transcription 3 (STAT3) signaling pathway,^40^ and exosome‐mediated paracrine miR‐34a‐5p CAF triggers oral cancer cells proliferation and metastasis.^41^ Chen et al revealed that exosome‐mediated transfer of miR‐93‐5p from CAFs led to radioresistance in CRC cells through the downregulation of FOXA1 and upregulation of TGF‐β3.^42^

### 2.3 TCDE and immune escape

Immune system displays a critical role in maintaining tumor development and progression. Exosomes from both non‐immune and immune cells participate in immune regulation, such as mediating T cell apoptosis, reducing NK‐ and T cells, and triggering the differentiation of myeloid cells to myeloid derived suppressor cells.^43^ To destroy the anti‐tumor immunity, tumor‐derived‐exosomes deliver the suppressive proteins and nucleic acids to immune cells including monocytes, macrophages, dendritic cells (DCs), natural killer cells, T cells, B cells and thus evading immune surveillance.^44^, ^45^ programmed death‐ligand 1 (PD‐L1) is one of these suppressive proteins that exist on the surface of exosomes.^46^ Exosomal PD‐L1 systemically tends to suppress the anti‐tumor immune response, and its genetic blockage promotes T cell activity in the draining lymph node resulting in the induction of systemic anti‐tumor immunity and memory.^47^ On this basis, investigations revealed that exosomal PD‐L1 can be upregulated by interferon‐γ, thereby suppressing CD8^+^T cells and inducing tumor growth.^48^ Besides, it has been revealed that PD‐L1‐induced immunosuppression is correlated with anti‐PD‐1 response, implying exosomal PD‐L1 can be applied as a biomarker for anti‐PD‐1 therapy.^48^ In addition, compared to soluble PD‐L1 which increases in healthy individuals with age, exosomal PD‐L1 provides alternative options for cancer diagnosis and prognosis prediction.^49^ In parallel, non‐small cell lung cancer (NSCLC) cell‐derived exosomal PD‐L1 fosters tumor growth through immune escape of identical mechanisms.^50^ Another study in HCC reported that tumor‐derived exosomes could promote the immune‐suppressive regulatory B cells (Bregs), a kind of B cells subsets.^51^

High mobility group box 1 (HMGB1), a kind of DNA‐binding nuclear protein, is expressed on exosome membranes. Interestingly, studies illustrated that Bregs infiltrated tumors, and T‐cell immunoglobulin mucin domain‐1 (TIM‐1) (a marker of Bregs)^+^Bregs exhibited enhanced suppressive activity against CD8^+^T cells. Researchers detected that the expansion of Bregs promoted the advanced disease stage through the exosomal‐derived HMGB1 activation.^52^ In melanoma, tumor exosomes harboring enriched hsa‐miR‐3187‐3p, hsa‐miR‐498, hsa‐miR‐122, hsa‐miR149, and hsa‐miR‐181a/b elicit the immune escape by regulating TCR signaling and TNFα secretion.^53^ Furthermore, Haderk et al discovered the protumorigenic skewing mechanisms related to PD‐L1 expression in CLL and concluded that CLL‐derived exosomes mediated the transfer of non‐coding RNAs to monocytes, leading to the immune escape.^54^

### 2.4 TCDE and epithelial‐mesenchymal transformation

Epithelial‐mesenchymal transformation (EMT) is a conserved process characterized as the loss of epithelial features and the possession of mesenchymal phenotype.^8^ Prior research generally demonstrates that EMT is a tumor‐associated process that may elicit tumor cell invasion, metastasis, and drug resistance. Zhang et al proved that hypoxic bone marrow‐derived MSCs (BMSCs)‐derived exosomes promoted tumor cell invasion and EMT.^55^ Additionally, using miR‐193a‐3p, miR‐210‐3p, and miR‐5100‐loading exosomes together exerts better diagnostic accuracy in patients with lung cancer metastasis compared with respective treatment.^55^ Exosomes secreted from cancer cells are implicated in inducing EMT in cancer development. Wang et al conducted the study on the correlation between cancer stem cells (CSC) from clear cell renal cell carcinoma and EMT. The outcome revealed that CSC exosomes initiated EMT through the transmission of MIR‐19b‐3p to CCSCC cells, which was related to the repressed expression of phosphatase and tensin homolog (PTEN). What's more, metastasis exosomes display stronger EMT effect.^56^ EMT occurs in melanoma cell‐derived exosomes as a consequence of the activation of MAPK pathway, thus leading to metastasis. Decreased let‐7, a miRNA modulator of EMT is detected, which may account for this phenomenon.^57^ Similar examples are shown in HCC‐derived exosomes. Highly metastatic HCC‐derived exosomes improve migration, chemotaxis, and invasion of low metastatic cells by transferring pro‐metastatic molecules. Abrogating the release of these exosomal factors through inhibition of Rab27a, a modulator of exosome secretion, promotes invasion of parental cells.^58^ Vps4A, functioning as a tumor suppressor through regulation of exosomal miRNAs in HCC, is demonstrated to mediate the plasma membrane localization and decrease exosome‐released β‐catenin, thus inhibiting EMT when it is overexpressed.^59^, ^60^

As mentioned above, CAFs act as abettors in tumor progression, including the induction of EMT.^61^ Yeon et al observed that MSC‐derived exosomes reduced CAFs and suppressed EMT, thereby inducing angiogenesis and maintaining vascular homeostasis, while cancer‐derived exosomes that transdifferentiate CAFs promoted EMT.^62^ Hu et al examined the elevated level of miR‐92a‐3p as a consequence of CAFs transferring exosomes to CRC cells. Additionally, increased miR‐92a‐3p activated Wnt/β‐catenin pathway and inhibited mitochondrial apoptosis, leading to EMT and thus promoting metastasis and chemoresistance.^63^ Another similar study shows the promotion of EMT in breast cancer caused by CAFs secreted exosomal microRNA‐181d‐5p and the underlying mechanisms is related to the downregulation of homeobox A5 (HOXA5) and caudal‐related homeobox 2 (CDX2).^64^ Studies regarding the correlation between CAFs and EMT have been performed, providing understandings on repressing tumor progression.

### 2.5 TCDE and pre‐metastatic niche

Pre‐metastatic niche in cancers is a prevalent precondition of metastasis. Liu et al elucidated it with six pro‐metastasis features including immunosuppression, inflammation, angiogenesis/ vascular permeability, lymphangiogenesis, organotropism, and reprogramming.^65^ There is an urgent need to seek novel and effective approaches targeting pre‐metastasis niche to make amends for the delayed detection and prevent further metastasis.

The consensus has been that exosomes participate in pre‐metastatic niche formation of cancers.^66^ Costa‐Silva et al found that pancreatic ductal adenocarcinomas (PDAC)‐derived exosomes were integrated with Kupffer cells, a kind of macrophage, to stimulate the fibronectin production and induce the formation of pre‐metastasis niche. Furthermore, macrophage migration inhibitory factors (MIF), which were released by macrophages in fibrotic microenvironment and enriched in PDAC‐exosomes, could prevent the niche formation when it was blocked.^67^ Zeng et al manifested the involvement of CRC‐derived exosomal miR‐25‐3p in promoting pre‐metastatic niche formation through mechanisms related to the angiogenesis and vascular permeability.^68^ Toll‐like receptor 3 (TLR3) are able to recognize inflammatory signals and promote tumor progression. Liu et al uncovered that deficiency of TLR3 in lung epithelial cells prevented the niche formation, while exosomal RNA reversed this process by activating TLR3, consequently recruiting Neutrophils and favoring the niche formation.^69^ Similarly, melanosomes foster tumor niche by reprogramming dermal fibroblast into CAFs through transferring miR‐211.^70^ Contrary to the aforementioned research, Plebanek et al discovered that non‐metastasic exosomes resulted in the inhibition of metastasis. It is identified that pre‐metastatic tumors release exosomes, simultaneously eliciting immune surveillance, resulting in tumor cell clearance at the pre‐metastatic niche.^71^

It has also been reported that melanoma‐derived exosomes facilitate the formation of pre‐metastatic niche via induction of vascular leakiness and pro‐vasculogenic phenotype of bone marrow progenitors.^72^ Another notable investigation based on previous exosome‐mediated metastasis theory revealed the crucial role of exosome integrin in promoting metastasis. Hoshino et al provided a rational of exosome integrin predicting organotropism of metastatic cells, which indicated the promising prospect of the application in diagnosis and treatment.^73^

### 2.6 TCDE and drug resistance

Acquired drug resistance is a troublesome obstacle that causes limited therapeutic response in cancer patients.^74^ Previous studies have documented that exosomes derived from tumor cells or related stromal cells mediate the drug resistance through the exosomal transfer of miRNAs.^75^ The miR‐21 transferred from macrophages to GC cells was detected to reduce cisplatin chemosensitivity and suppress apoptosis.^76^ However, exosome‐transmitted miR‐128‐3p overexpression displays enhanced chemosensitivity in oxaliplatin‐resistant advanced CRC through altering target gene expression, while lower expression of miR‐128‐3p leads to poor drug response.^77^ In glioblastoma, exosomal transfer of miR‐15a and miR‐1238 was substantiated to contribute to chemotherapeutic‐resistance.^78^, ^79^ In multidrug‐resistant HCC cells, elevated exosomal miRNA‐32‐5p and reduced PTEN are associated with poor prognosis. Additionally, miRNA‐32‐5p targeting PTEN induces multidrug resistance via the PI3K/Akt pathway.^80^ EphA2 is overexpressed in tumors and has been recognized as a pivotal mediator of chemoresistance. In pancreatic cancer, exosome‐mediated EphA2 transfer is revealed to perform chemoresistance transmission between variable gemcitabine resistance cell lines.^81^ Dorayappan et al demonstrated that exosomes were greatly secreted under hypoxic condition, and these hypoxia‐induced exosomes increased cisplatin efflux, augmenting metastasis and chemotherapy resistance in ovarian cancer.^82^ Another investigation in ovarian cancer has shown that exosomal miR‐1246 is potent effector in acquiring chemoresistance via modulating Cav1and p‐gp interaction.^83^

In addition, the expression of drug targets on exosomes, including PD‐L1, CD20, and CTLA‐4, may be a novel mechanism for drug resistance. These molecules carried on exosomes can deplete the specific drugs or antibodies to waken the therapeutic effects.^46^ Yang et al have proved that the accumulation of exosomal PD‐L1 in TME induced therapeutic resistance, and the successful inhibition of these exosomes’ secretion could recover the efficacy of anti‐PD‐1 therapy.^84^

## 3 ROLE OF EXOSOMES FROM OTHER CELLS IN TME FOR TUMOR PROGRESSION

### 3.1 Mesenchymal stem cell‐derived exosomes

MSCs are considered as crucial regulators of TME and control multiple aspects of cancer progression, including tumorigenesis, angiogenesis, and metastasis.^85^ It has been reported that the interactions between MSCs and tumor cells, predominately mediated by exosomes, is the key performer of MSCs functions. On the one hand, exosomes derived from tumor cells transfer biologically active proteins and nuclear acids to MSCs, co‐opting MSCs and transforming them into tumor supportive types. On the other hand, MSCs reprogrammed by tumor exosomes become avid producers of their own exosomes, secreting various factors accumulating cancer progression (Table 2).^2^, ^86^

**TABLE 2:** Role of exosomes from other cells in TME for tumor progression

Cancer type	Cargo	Function	Mechanism	Reference
**Mesenchymal stem cells**				
Lung cancer	miR‐193a‐3p miR‐210‐3p miR‐5100	Promote cancer cell invasion and EMT	Activate STAT3 signaling pathway	^55^
Glioblastoma	miR‐1587	Increase the proliferation and clonogenicity of tumor‐initiating stem‐like cells, lead to greater tumor burden and decreased survival immune regulation and inhibit B‐lymphocytes functions	Downregulation of NCOR1	^87^
Breast cancer	/	Promote cell growth and protect tumor cells from chemotherapeutic drug‐induced apoptosis in vitro	Activation of Hippo signaling pathway	^89^
Prostate cancer	miR‐205	Repress cell proliferation, invasion and migration, impede cancer progression	Inhibit RHPN2	^90^
Pancreatic Cancer	miR‐126‐3p	Inhibit the proliferation, invasion, and metastasis of pancreatic cancer cells, and promote apoptosis; suppress cancer development	Target ADAM9	^91^
Glioblastoma	miR‐133b	Suppress cancer development	Inhibit the Wnt/β‐catenin signaling pathway	^92^
**Fibroblast**				
Pancreatic cancer	/	Promote proliferation and drug resistance	Increased snail	^94^
Colorectal cancer	miR‐92a‐3p overexpression	Promote metastasis and chemotherapy resistance	Activate Wnt/β‐catenin pathway, inhibit mitochondrial apoptosis	^63^
Gastric cancer	miR‐522	Decrease chemo‐sensitivity and inhibit ferroptosis	Suppress ALOX15 expression and lipid‐ROS accumulation	^95^
Colorectal cancer	lncRNAs H19	Promote the stemness and chemoresistance	Activate the β‐catenin pathway	^96^
**Macrophage**				
Colorectal cancer	miR‐21‐5p miR‐155‐5p	Induce cancer cell migration and invasion	Downregulate BRG1 expression	^98^
Pancreatic ductal adenocarcinoma	miR‐501‐3p	Promote tumor progression	Target TGFBR3 and activate TGF‐β signaling pathway	^99^
Gastric cancer	miR‐21	Contribute to cisplatin resistance	Activate PI3K/AKT signaling pathway and inhibit cell apoptosis	^76^
Gastric cancer	Functional apolipoprotein E	Remodel the cytoskeleton‐supporting migration	Activate PI3K‐Akt signaling pathway	^100^

Symbol “/” represents the unknown cargo.

Zhang et al found that exosomes released by bone marrow‐derived MSCs (BMSCs) could promote invasion of lung cancer cells by activating STAT3 signaling pathway. The microRNAs, including miR‐193a‐3p, miR‐210‐3p, and miR‐5100, transferred by exosomes from BMSCs to epithelial cancer cells exhibited diagnostic accuracy that discriminate cancer patients from non‐cancerous controls.^55^ Additionally, MSC‐derived exosomes from glioma tissues increase the proliferation and clonogenicity of tumor‐initiating stem‐like cells, leading to greater tumor burden and decreased survival.^87^ Moreover, MSC‐derived exosomes are implicated in immune regulation and inhibit the functions of B‐lymphocytes by affecting the expression of specific mRNAs.^88^ In a recent study, Wang et al demonstrated that exosomes secreted by MSC‐differentiated adipocytes promote breast cancer cell growth and protect tumor cells from chemotherapeutic drug‐induced apoptosis in vitro. The inhibition of exosomes attenuates the tumor‐promoting effects of adipocytes.^89^

While MSC‐derived exosomes primarily exert pro‐tumor effects in cancer development, studies also reported the tumor suppressive activities of exosomes secreted by MSCs. Jiang and colleagues showed that MSC‐derived exosomes carrying miR‐205 could repress prostate cancer cell proliferation, invasion, and migration, thus impeding cancer progression through inhibiting rhophilin Rho GTPase binding protein 2 (RHPN2).^90^ Similarly, MSC‐derived exosomal miR‐126‐3p suppresses the development of pancreatic cancer by targeting ADAM9.^91^ In another study, Xu et al found that exosomes containing miR‐133b released by MSCs attenuated glioma development via inhibiting the Wnt/β‐catenin signaling pathway.^92^ Collectively, the diverse cargos transferred by MSC‐derived exosomes are the predominant determinant of their effects in tumor progression, and MSCs also varies dependent on local TME.^85^

### 3.2 Fibroblast‐derived exosomes

Exosomes released by tumor cells promote tumorigenesis through activation of CAFs, which actively remold TME and sustain a cancer permissive state. Conversely, CAFs‐derived exosomes also significantly affect tumor cells and facilitate tumor progression.^93^ A number of studies have demonstrated that CAFs‐derived exosomes are involved in tumor cell proliferation, migration, metastasis, and drug resistance.^63^, ^94^, ^95^, ^96^ For example, fibroblast exosomes have been shown to enhance the survival and proliferation of pancreatic cancer cells, while inhibition of exosome release by GW4869 significantly reduces survival of cancer epithelial cells.^94^ Hu et al found that exosomes secreted by CAFs are transferred to CRC cells and lead to overexpression of miR‐92a‐3p, activating Wnt/β‐catenin pathway and inhibiting mitochondrial apoptosis, thus promoting metastasis and chemotherapy resistance.^63^ In another study, exosomal miR‐522 derived from CAFs suppresses ALOX15 expression and lipid‐ROS accumulation in cancer cells, decreasing chemo‐sensitivity and inhibiting ferroptosis, a novel mode of non‐apoptotic cell death.^95^ CAFs‐derived exosomes have also been shown to contain long non‐coding RNAs (lncRNAs) that promote the stemness and chemoresistance of CRC.^96^

### 3.3 Macrophage‐derived exosomes

Macrophages within the TME play a critical role in cancer development, which promote cancer initiation and progression by facilitating cancer cell proliferation, migration, angiogenesis, and immunosuppression.^97^ Macrophage‐derived exosomes orchestrate their communication with cancer cells, significantly affecting the malignant progression.

It has been shown that M2 macrophage‐derived exosomes transfer miR‐21‐5p and miR‐155‐5p to CRC cells, inducing cancer cell migration and invasion.^98^ In addition, exosomes released by macrophages promote progression of PDAC by targeting transforming growth factor beta receptor 3 (TGFBR3), a tumor suppressor gene, and activating TGF‐β signaling pathway.^99^ It has also been found that M2 macrophage‐derived exosomes contribute to cisplatin resistance in GC cells through delivery of miR‐21, which activates PI3K/AKT signaling pathway and inhibits cell apoptosis.^76^ In another study, the authors demonstrated that macrophage‐derived exosomes transfer functional apolipoprotein E to GC cells and promote the migration of cancer cells.^100^

## 4 APPLICATION OF EXOSOMES IN DIAGNOSIS

In most cases, the poor therapeutic response and prognosis in cancer patients are due to the late detection of diseases, which is largely attributed to the lack of appropriate biomarkers that serve as efficient diagnostic tools.^101^ Therefore, considerable attention has been drawn to identifying and validating disease‐specific molecules that could be utilized for cancer diagnosis and prediction (Figure 3). Exosomes containing various proteins, lipids, nucleic acids, and metabolites are considered as the rich source of potential biomarkers with unparalleled advantages.^102^ First, almost all cells secrete exosomes, and the multiple cargos carried by exosomes reflect the physiological status of their parental cells.^103^ Second, apart from being secreted by different cells in vivo, exosomes also widely exist in various body fluids, such as blood, urine, and saliva.^103^ Thus, exosomes are ideal noninvasive biomarkers and easily accessible for clinical detection. Third, due to the unique structure of exosomes, proteins and nucleic acids could be well protected by the lipid bilayer, and important information about disease characteristics is preserved.^101^

miRNAs are one of the biological cargos in exosomes that participate in multiple aspects of cancer progression. Emerging evidence suggests that miRNAs are potential biomarkers for cancer diagnosis and prediction due to their stable properties and extreme abundance in exosomes (Table 3).^19^ Importantly, with the development of specific and sensitive quantitative techniques, exosomal miRNAs can be detected in small volume samples with high accuracy.^103^ Clinical studies have shown that miR‐126, a tumor suppressor and metastasis inhibitor, is downregulated in NSCLC patients. Additionally, miR‐126 is equally distributed between exosomes and exosome‐free serum fractions in healthy people while mainly present in exosomes of NSCLC patients, suggesting the potential of miR‐126 as a reliable disease biomarker.^104^ Furthermore, Jin et al have identified AC‐ and SCC‐specific miRNAs as diagnostic biomarkers for early stage NSCLC by using next‐generation sequencing. The validated miRNAs with diagnostic accuracy such as AC‐specific miR‐181‐5p, miR‐30a‐3p, miR‐30e‐3p, and miR‐361‐5p, and SCC‐specific miR‐10b‐5p, miR‐15b‐5p, and miR‐320b could be promising indicators in the diagnosis of NSCLC.^105^ It has also been reported that the expression level of exosomal miR‐25‐3p is significantly elevated in CRC patients with metastasis than those without metastasis. Mechanism analysis has shown that miR‐25‐3p regulates the expression of VEGFR2, ZO‐1, occludin, and Claudin5 in endothelial cells by targeting KLF2 and KLF4, promoting angiogenesis and pre‐metastatic niche formation.^68^ Interestingly, Cheng et al have demonstrated that the secretion of colorectal CSC exosomes carrying biomarker miR‐146a‐5p is dependent on the expression of RAB27B by activation of β‐catenin/Tcf‐4.^106^ In addition, Xue et al identified the differentially expressed exosomal miRNAs in cholangiocarcinoma and gallbladder carcinoma patients with high‐throughput small RNA sequencing. After a larger‐scale validation, they found that miR‐96‐5p, miR‐151a‐5p, miR‐191‐5p, and miR‐4732‐3p were significantly increased in the exosomes of cholangiocarcinoma patients, while miR‐151a‐5p was increased in the exosomes of gallbladder carcinoma patients.^107^ In summary, although a multitude of miRNAs have been identified and validated as potential diagnostic markers for cancer in the basic research studies, much clinical data are also required to confirm their diagnostic effects.^19^

**TABLE 3:** Exosomal diagnostic biomarkers for detection of tumor progression

Cancer type	Biomarker	Source of exosomes	Potential mechanisms	Isolation methods	Reference
Non‐small cell lung cancer (NSCLC)	miR‐126↓	Serum	Suppress tumorigenic function by targeting EGFL7	Ultracentrifugation	^104^
Non‐small cell lung cancer‐ adenocarcinoma (NSCLC‐AC)	miR‐181‐5p↑ miR‐30a‐3p↓ miR‐30e‐3p↓ miR‐361‐5p↑	Plasma	Not mentioned	Ultracentrifugation and immunoaffinity magnetic beads	^105^
Non‐small cell lung cancer‐ squamous cell carcinoma (NSCLC‐SCC)	miR‐10b‐5p↓ miR‐15b‐5p↓ miR‐320b↑	Plasma	Not mentioned	Ultracentrifugation and immunoaffinity magnetic beads	^105^
Colorectal cancer (CRC)	miR‐25‐3p↑	CRC cells	Regulate VEGFR2, ZO‐1, occludin and Claudin5 by targeting KLF2 and KLF4	Ultracentrifugation	^68^
Colorectal cancer (CRC)	miR‐146a‐5p↑	CRC stem cells	Target Numb in recipient CRC cells	Ultracentrifugation	^106^
Cholangiocarcinoma (CCA)	miR‐96‐5p↑ miR‐151a‐5p↑ miR‐191‐5p↑ miR‐4732‐3p↑	Plasma	Not mentioned	exoEasy maxi kit (QIAGEN)	^107^
Bladder cancer (BC)	lncRNA‐UCA1↑	5637 cells and serum	Promote tumor growth and progression through EMT	Ultracentrifugation	^108^
Bladder cancer (BC)	lncRNA PTENP1↓	Plasma	Mediate PTEN expression by competitively binding to microRNA‐17, inhibit cell migration, invasion and induce apoptosis	Exoquick exosome precipitation solution (System Biosciences)	^109^
Bladder cancer (BC)	MALAT1↑, PCAT‐1↑ SPRY4‐IT1↑	Urine	Not mentioned	Not mentioned	^110^
Gastric cancer (GC)	lncUEGC1↑ lncUEGC2↑	Plasma, epithelial cells; GC cells	Not mentioned	Ultracentrifugation and discontinuous iodixanol gradient methods.	^111^
Gastric cancer (GC)	lncRNA HOTTIP↑	Serum	Positively associate with invasion depth and TNM stage	Ultracentrifugation	^112^
Glioblastoma (GBM)	lncSBF2‐AS1↑	GBM cells	Confer temozolomide chemoresistance	Ultracentrifugation	^113^
Pancreatic ductal adenocarcinomas (PDAC)	circ‐PDE8A↑	PDAC cells	Promote PDAC cells invasion by upregulating MET	TRIzol LS (Thermo, Carlsbad, CA)	^116^
Pancreatic ductal adenocarcinomas (PDAC)	circ‐IARS↑	PDAC cells	Regulate endothelial monolayer permeability	Total Exosome Isolation Kit (Thermo, Carlsbad, CA)	^117^
Gastric cancer (GC)	circ‐KIAA1244↓	GC tissues, plasmas, and cells	Not mentioned	Hieff Quick exosome isolation kit (for serum/plasma, Shanghai, 41202ES30)	^118^
Gastric cancer (GC)	circNRIP1↑	GC cells	Via the AKT1/mTOR pathway, function as a microRNA‐149‐5p sponge	Exosome isolation reagents (4,478,359, Invitrogen)	^119^
Head and neck squamous cell carcinomas (HNSCCs)	PD‐L1↑	Plasma	Induce immune escape and lead to immunotherapy resistance	Ultracentrifugation	^120^
Pancreatic cancer (PC)	ZIP4↑	PC cells	Enhance cell proliferation, migration and invasion abilities	SBI ExoQuick‐TC Kit (System Biosciences, Mountain View, CA)	^121^

Arrow ↑/↓ represents the increased/decreased expression of biomarker.

In addition to miRNAs, lncRNAs are also attractive candidates in the development of sensitive and specific biomarkers for cancer diagnosis and prediction. These lncRNAs are enriched in exosomes and stable in blood, reflecting the cell status that they origin. Additionally, the different expression level of lncRNAs in tumor tissues and healthy organs suggests their potential clinical application as cancer‐specific biomarkers. For instance, the expression level of lncRNA‐UCA1 is significantly higher in exosomes of BC patients than in healthy donors and could be used for the clinical detection of BC.^108^ While in another study, Zheng et al found that lncRNA PTENP1 was significantly reduced in BC tissues, and normal cell derived‐exosomal PTENP1 could be transferred to cancer cells, impeding cancer progression by inhibiting cell migration, invasion, and inducing apoptosis.^109^ Moreover, reports have established that a urinary exosome‐derived lncRNA panel (MALAT1, PCAT‐1, and SPRY4‐IT1) could be used as noninvasive diagnostic and prognostic biomarker of BC with considerable clinical value. The upregulation of PCAT‐1 and MALAT1 indicates poor recurrence‐free survival.^110^ Tumor‐originated exosomal lncUEGC1 is expressed at a high level in stage I GC patients and GC cells. Notably, it exhibits higher diagnostic accuracy than carcinoembryonic antigen in discriminating early GC patients from healthy individuals and late stage patients.^111^ In addition, Zhao el al demonstrated that the expression level of lncRNA HOTTIP is significantly correlated with invasion depth, tumor‐node‐metastasis (TNM) stage, and overall survival of GC patients, functioning as an independent prognostic factor.^112^ Recent studies have reported that exosomal lncSBF2‐AS1 confers chemoresistance to glioblastoma cells, and high levels of lncSBF2‐AS1 are associated with poor response to temozolomide treatment in glioblastoma patients.^113^

In recent years, circular RNAs (circRNAs) are emerged as novel biomarkers for cancer diagnosis due to their universal existence in eukaryotic cells and tissue‐ and developmental stage‐specific expression patterns.^114^ Accumulating evidence indicates that circRNAs are regulators of parental gene transcription and exhibit diverse biological functions in the progression of cancer.^115^ Exosomal circ‐PDE8A derived from plasma of PDAC patients has been reported to promote tumor invasion by upregulating MET (MET proto‐oncogene, receptor tyrosine kinase). The expression level of circ‐PDE8A is positively correlated with lymphatic invasion, TNM stage and negatively correlated with survival rate, acting as a promising indicator for PDAC diagnosis and prognosis.^116^ In addition, Li et al found that circ‐IARS secreted by PDAC tumor cells could be transferred to human microvascular vein endothelial (HUVECs) cells and facilitate tumor invasion and metastasis.^117^ By screening circRNA expression profiles for GC, Tang and colleagues identified a novel circulating biomarker circ‐KIAA1244 in the detection of GC. Clinical data analysis demonstrated that low expression level of circ‐KIAA1244 indicated lymphatic metastasis and a shorter overall survival time.^118^ A recent study revealed that circNRIP1 delivered by exosomes promoted GC progression via the AKT1/mTOR pathway, functioning as a microRNA‐149‐5p sponge.^119^

Finally, proteins within exosomes also possess clinical potential as diagnostic markers. It has been reported that PD‐L1 levels in exosomes are correlated with progression of head and neck SCCs (HNSCCs), and exosomal PD‐L1 is involved in inducing immune escape that leads to resistance of immunotherapy.^120^ Studies have demonstrated that ZIP4, a membrane‐located zinc ion transporter, could be carried by exosomes to tumor cells and promotes pancreatic cancer growth. The expression level of ZIP4 varies in different stages of pancreatic cancer, and high ZIP4 expression is correlated with worse survival rates.^121^ Although numerous basic studies have shown strong relationship between exosome‐associated proteins and cancer progression, the high heterogeneity of exosome‐associated proteins and the limited isolation methods significantly hinder their clinical practice. In addition, further clinical research are also required to verify their application in the diagnosis of cancer or monitoring during treatment.^122^

Although exosomes serving as biomarkers hold great promise in cancer diagnosis and prognosis prediction, most of research is currently in the early stage of exploration and lacks of clinical practice.^103^ In addition, given the predominance of non‐tumor derived exosomes in human body fluids such as platelet‐derived exosomes in blood plasma, the accurate separation, identification, and purification of exosomes are still facing considerable challenges. Further research on exosomal proteomics and novel detection methods will undoubtedly accelerate the clinical application of exosomes in cancer diagnosis.^123^

## 5 APPLICATION OF EXOSOMES IN THERAPY

### 5.1 Exosome‐based immunotherapy

In recent years, immunotherapy is emerged as critically important foci in the field of cancer research, and exosomes have attracted enormous attention in the development of novel immunotherapeutic modalities.^124^ Exosomes are proved to be vital mediators of immune regulation in TME.^125^ It has been showed that exosomes play significant roles in transfer of antigen and signals to antigen‐presenting cells directly or indirectly, thereby promoting the activation of immunity.^43^ What's more, the intrinsic immunomodulatory capability of exosomes endows them with high clinical application serving as cancer therapeutic vaccines that are able to induce adaptive and innate immunosurveillance.^124^ An impressive study by Lu et al revealed that exosomes derived from α‐fetoprotein expressing dendritic cells (DEX_AFP_) could trigger potent antigen‐specific antitumor immune responses and result in significant tumor growth retardation. Moreover, DEX_AFP_ improved the TME and prolonged the survival rates in three different HCC mouse models with antigenic and pathological heterogeneity.^126^ Importantly, the loading of exosomes to DCs can augment the vaccine efficacy, which is even superior to lysate‐loaded DCs.^127^ In addition, the combination of exosome‐based therapeutic cancer vaccines with other therapeutic modalities, such as chemotherapy and photothermal therapy, may enhance the efficacy of immunotherapy and exert stronger antitumor effect. Morishita and colleagues designed an exosome‐based tumor antigens‐adjuvant co‐delivery system that exosomes were modified with CpG DNA (CpG‐SAV‐exo). CpG‐SAV‐exo induced tumor antigen‐specific immune response more effectively than simple coadministration of exosomes and CpG DNA and significantly inhibited tumor growth.^128^

### 5.2 Exosome‐based drug delivery

With momentous progress in elucidating the biological and functional properties of exosomes, these natural nano‐vehicles with good biocompatibility, stability, and safety are recognized as compelling candidate platforms for drug delivery.^125^ Meanwhile, exosomes display exquisite target‐homing specificity and can be preferentially transferred to tumor cells with reduced side effects.^122^ Exosome‐based nanoplatforms have been employed to encapsulate and transfer multiple functional molecules in cancer treatment, such as chemotherapeutic drugs, proteins, and nucleic acids, and yielded encouraging preclinical results.^124^ For instance, doxorubicin has been successfully encapsulated in exosomes to ameliorate their biodistribution.^129^ Exosomes can also be deployed in delivering natural phytochemical compounds that with poor solubility, such as curcumin, to improve their bioavailability and accelerate their clinical translation.^130^ Recently, with the rapid development of gene therapy, exosomes hold great promise in the delivery of nucleic acid macromolecules. Kamerkar et al developed an exosome­based nanoplatform carrying short interfering RNA or short hairpin RNA for specific targeting of oncogenic KRAS. The exosomal formulation displayed enhanced circulating stability due to CD47‐mediated protection from phagocytosis.^131^

## 6 CONCLUSION

The past two decades have witnessed the admirable progress of exosomal science and therapeutics. These natural cell‐derived vesicles possessing unique structural characteristics are regarded as key mediators in intercellular communications and play an important role in cancer progression. The thorough understanding of these specific mechanisms by which exosome‐based communication regulates the malignant behavior of cancer cells and stromal cells in TME can make substantial contributions to the development of next‐generation exosomal therapies. In addition, as one of the most distinguishing hallmarks of exosomes, the variable cargo contents within exosomes carrying abundant, specific, and sensitive biological information are emerged as valid biomarkers in cancer diagnosis and prognosis. The enticing results of exosome based‐therapeutics and diagnosis in experimental settings have granted them a fast‐tracking into clinic.

With the rapid development of nanotechnology, exosomes are heralded as attractive candidates in the establishment of novel nanoplatforms for cancer treatment. Compared with conventional synthetic nanocarriers, these exosomal nanoformulations display fascinating advantages in drug delivery and are being rampantly developed. However, some obstacles still lay ahead for the optimal translation of these promising results into clinic, such as the technical challenges of exosome isolation, purification, and identification; and tremendous efforts are required to overcome the existing limitations to achieve clinical benefit.

## CONFLICT OF INTEREST

The authors declare that there is no conflict of interest that could be perceived as prejudicing the impartiality of the research reported.

## AUTHOR CONTRIBUTIONS

Wen‐Jie Gu and Yi‐Wen Shen searched the literature and wrote the manuscript. Li‐Jun Zhang and Hong Zhang gave some supplements of current literature. Dale G. Nagle checked the grammar and polished the language. Xin Luan and San‐Hong Liu made conceptual revision and checked the final paper to be published.

## ETHICS APPROVAL STATEMENT

Not applicable.

## Data Availability

Not applicable.
